# Evaluating the impact of sickle cell disease on COVID-19 susceptibility and severity: a retrospective cohort study based on electronic health record

**DOI:** 10.3389/fepid.2023.1241645

**Published:** 2023-09-12

**Authors:** Jiajun Luo, Johnny Powell, Sage Ross, Julie Johnson, Christopher O. Olopade, Jayant Pinto, Karen Kim, Habibul Ahsan, Briseis Aschebrook-Kilfoy

**Affiliations:** ^1^ Department of Public Health Sciences, University of Chicago, Chicago, IL, United States; ^2^ Institute for Population and Precision Health, University of Chicago, Chicago, IL, United States; ^3^ Comprehensive Cancer Center, University of Chicago, Chicago, IL, United States; ^4^ Center for Research Informatics, University of Chicago, Chicago, IL, United States; ^5^ Department of Medicine, University of Chicago, Chicago, IL, United States; ^6^ Department of Surgery, University of Chicago, Chicago, IL, United States

**Keywords:** causal inference, sickle cell disease, sickle cell trait, propensity score, ARDS, pneumonia

## Abstract

**Background:**

Sickle cell trait/disease (SCT/SCD) are enriched among Black people and associated with various comorbidities. The overrepresentation of these characteristics prevents traditional regression approach obtaining convincing evidence for the independent effect of SCT/SCD on other health outcomes. This study aims to investigate the association between SCT/SCD and COVID-19-related outcomes using causal inference approaches that balance the covariate.

**Methods:**

We leveraged electronic health record (EHR) data from the University of Chicago Medicine between March 2020 and December 2021. Demographic characteristics were retrieved. Medical conditions were identified using ICD-10 codes. Five approaches, including two traditional regression approaches (unadjusted and adjusted) and three causal inference approaches [covariate balancing propensity score (CBPS) matching, CBPS weighting, and CBPS adjustment], were employed.

**Results:**

A total of 112,334 patients were included in the study, among which 504 had SCT and 388 SCD. Patients with SCT/SCD were more likely to be non-Hispanic Black people, younger, female, non-smokers, and had a diagnosis of diabetes, heart failure, asthma, and cerebral infarction. Causal inference approaches achieved a balanced distribution of these covariates while traditional approaches failed. Across these approaches, SCD was consistently associated with COVID-19-related pneumonia (odds ratios (OR) estimates, 3.23 (95% CI: 2.13–4.89) to 2.57 (95% CI: 1.10–6.00)) and pain (OR estimates, 6.51 (95% CI: 4.68–9.06) to 2.47 (95% CI: 1.35–4.49)). While CBPS matching suggested an association between SCD and COVID-19-related acute respiratory distress syndrome (OR = 2.01, 95% CI: 0.97–4.17), this association was significant in other approaches (OR estimates, 2.96 (95% CI: 1.69–5.18) to 2.50 (95% CI: 1.43–4.37)). No association was observed between SCT and COVID-19-related outcomes in causal inference approaches.

**Conclusion:**

Using causal inference approaches, we provide comprehensive evidence for the link between SCT/SCD and COVID-19-related outcomes.

## Introduction

The COVID-19 pandemic has impacted millions of people and imposed unprecedented burdens on healthcare system around the world since its outbreak in the early 2020. Ample evidence has shown that individuals with preexisting medical conditions, such as hypertension, diabetes, chronic obstructive pulmonary disease (COPD), and chronic kidney disease, are more likely to experience severe COVID-19 outcomes (1). Meanwhile, the COVID-19 pandemic also highlights the racial health disparities in the US. According to the surveillance data from the Centers for Disease Control and Prevention as of July, 2022, when compared to non-Hispanic White people, non-Hispanic Black people are 2.2 times more likely to be hospitalized due to COVID-19, and 1.7 times more likely to die from COVID-19 (2). Although social determinants, such as structural racism and lower socio-economic status (SES), certainly impact this disparity (3), it is possible that other physiological constraints prevalent among minority groups exacerbate COVID-19 outcomes (4).

Sickle cell trait/disease (SCT/SCD) is an inherited red blood cell abnormality caused by genetic mutations in the β-globin chain gene that predominantly occurs among persons of African ancestry (5). It is estimated that 8% to 10% of non-Hispanic Black people live with SCT and 1 in 400 non-Hispanic Black people are affected by SCD in the US (5), while the prevalence of SCT/SCD is rare among other racial/ethnic groups (5). Individuals with SCT often have the same quality of life as the general population and do not necessarily have SCD and SCD-associated comorbidities (6). However, the sickle hemoglobin-containing red blood cells still expose individuals with SCT/SCD to a variety of adverse health impacts including organ damage and early mortality, especially in low oxygen environments (6–10). Since COVID-19 is characterized by hypoxia (11), and SCT and SCD are both hypothesized to worsen COVID-19 outcomes and contribute to the racial health disparities during the pandemic, investigation of this topic is warranted (4).

As SCT/SCD is usually accompanied with a wide range of comorbidities and enriched in non-Hispanic Black communities, traditional analytical methods may overestimate the adverse impact of SCT/SCD on COVID-19 outcomes because of difficulty to disentangle the roles of comorbidities and demographic characteristics from SCT/SCD. Patients with SCT/SCD can experience various pulmonary complications due to the effects of sickling on blood vessels and oxygen transport, while respiratory system is a major target of COVID-19. For example, SCT/SCD can increase the risk and severity of pneumonia or acute chest syndrome (ACS) due to various factors related to the underlying physiological changes caused by the disease including impaired immune function, vaso-occlusive events, and chronic inflammation (12). Meanwhile, COVID-19 primarily affects the lungs and can cause a range of symptoms, from mild respiratory issues to severe pneumonia (13). In the context of the COVID-19 pandemic, patients with SCT/SCD might be at an increased risk of severe outcomes if they contract the virus, including the potential for developing more severe forms of COVID-19-related respiratory complications, but the overrepresentation of certain demographic and clinic characteristics among patients with SCT/SCD prevents researchers investigating this question.

Recent development in causal inference approaches provides powerful tools to estimate the true association and even establish causality in this complex situation for SCT/SCD (14). As suggested by methodologists, causal inference approaches, which are more robust to violation of assumptions, are able to build a pseudo-population of the same demographic and clinic characteristics for causal comparison (15). In this study, using a large retrospective cohort based on the electronic health records (EHR) at the University of Chicago Medicine (UCM) during the pandemic, we applied traditional as well as causal inference approaches to estimate the association between SCT/SCD and COVID-19 outcomes, aiming to provide evidence of the causal link and illustrate the applications of causal inference approaches in this complex situation.

## Methods

### Study population

The study population consists of adult patients (age ≥18) who visited UCM in both inpatient and outpatient settings from March 2020 through December 2021. The data were retrieved in May, 2022. All UCM patients tested for COVID-19 during the time period and the test results were used to determine COVID-19 status and related outcomes. We obtained demographic, comorbidity, laboratory, UCM treatment, and outcome data from the EHR. Patients without valid EHR were excluded. Diagnoses of all diseases were recorded using ICD-10 codes in EHR. Demographic characteristics obtained from EHR included age group (≤35, 36–50, 51–65, >65), body mass index (BMI) group (<18.5, 18.5–25, 25.1–30, 30.1–40, >40, missing), race (non-Hispanic White, non-Hispanic Black, Hispanic, other, missing/unknown), sex (female, male, missing/unknown), and smoking history (never, current, or passive smoking, quit smoking, missing/unknown).

This study was approved by the University of Chicago Biological Sciences Division Institutional Review Board with a waiver of consent for use of de-identifiable data.

### SCT/SCD and comorbidities

The SCT/SCD status was identified by the presence of sickle cell ICD-10 diagnosis codes in the EHR. SCT was determined by ICD-10 code D57.3, while SCD was determined by ICD-10 codes D57.0, D57.1, D57.2, and D57.4 any time before COVID-19 diagnosis.

Diagnoses of preexisting comorbidities were also retrieved based on ICD-10 codes from EHR for all patients, including hypertension (I10 and I16), type 2 diabetes (E11), chronic kidney disease (N18), heart failure (I50), asthma (J45), COPD (J44), chronic ischemic heart disease (I25), and cerebral infarction (I63).

### COVID-19 and related outcomes

All patients who visited UCM were required to undergo COVID-19 PCR testing during the study period with the Roche assay (Cobas). COVID-19-related ICU admission and mortality were determined based on EHR within 2 weeks after COVID-19 diagnosis.

Other COVID-19-related outcomes were identified using ICD-10 codes in EHR, including acute respiratory distress syndrome (ARDS, J96), pneumonia/ACS (J12, J16, J18, D57.01, D57.811, and D57.411), pain (R07, R10, R52, R27, M25.5, M54, M79.6, M79.1, D57.00, D57.219, D57.419, and D57.819), shortness of breath or hypoxia (R06.00, R06.02, and R09.02), and venous thromboembolism and pulmonary embolism (VTE/PE, I26, I81, and I82). These complications were limited to within 2 weeks from positive COVID-19 test to ensure that they were specific to the infection.

### Statistical analysis

We firstly compared the distributions of basic demographic characteristics in our study population with the data from the US National Health and Nutrition Examination Survey (NHANES) 2017 to March 2020 pre-pandemic, which represents general US population. SCT/SCD status was the exposure of interest in this study. We analyzed SCT and SCD separately in the statistical models, with a goal to evaluate the distinct impacts between SCT and SCD on COVID-19 outcomes. All the variables used in the analysis were treated as categorical variables.

We fitted five different statistical models to evaluate the relationship between SCT/SCD and COVID-19 outcomes, two traditional regression models and three causal inference models. More details about these approaches can be found in the supplemental materials.

The first model was a traditional logistic regression model without adjustment for any covariates, giving the crude odds ratio (OR) and 95% confidence interval (CI) between SCT/SCD status and COVID-19-related outcomes.

The second model was a traditional logistic regression model with adjustment for aforementioned comorbidities and demographic characteristics, including age, BMI, race, sex, smoking history, hypertension, type 2 diabetes, chronic kidney disease, heart failure, asthma, COPD, chronic ischemic heart disease, and cerebral infarction, giving the traditional adjusted OR and 95% CI. In this statistical model, missing values in covariates were addressed using multiple imputation assuming missing at random (16). Ten complete datasets were generated and analyzed. The method proposed in early studies were used to combine these results (17).

The remaining three causal inference approaches required the estimation of propensity score as the first step. In this study, we used the covariate balancing propensity score (CBPS) method to calculate the propensity scores (18, 19). We modeled the SCT/SCD status on the same set of covariates used in the second statistical model in CBPS. Missing values in these covariates were treated as an independent category when computing CBPS. Compared to the conventional propensity score generated from logistic regression, the CBPS method concurrently maximizes the covariate balance and the SCT/SCD status prediction (18, 19). CBPS generated from this step was used in the following statistical models.

The third statistical model was matching based on CBPS. We employed optimal matching algorithm to perform 1:1 matching between patients with and without SCT/SCD (20). We checked the covariate balance in the matched pseudo-population, and if covariate balance was achieved [absolute standardized mean difference (ASMD) <0.1, see supplemental methods] (21); then we fitted an unadjusted conditional logistic regression model which takes matching into account to estimate the OR and 95% CI for SCT/SCD status in relation to COVID-19 outcomes among the matched pseudo-population (22).

The fourth statistical model was weighting based on CBPS. An inverse propensity score weight was generated for each patient as suggested by Imai and Ratkovic (19). We checked the covariate balance in the matched pseudo-population, and if covariate balance was achieved (ASMD <0.1, see supplemental methods) (21); then we fitted a weighted univariate generalized estimating equation model with an independent working correlation as suggested by Hernan and Robins to obtain the OR and 95% CI for SCT/SCD status in relation to COVID-19 outcomes among the weighted pseudo-population (23).

The fifth statistical model was a logistic regression model in which COVID-19 outcomes were regressed on SCT/SCD status and CBPS, which has been shown sufficient to remove confounding bias. A linear term and a quadratic term of CBPS were simultaneously included in the model. Compared to matching and weighting where the causal analysis is complete after fitting the regression model, the causal analysis in regression model adjusted for propensity score is conducted on the counterfactuals predicted by the model (24). More details can be found in the supplemental material. The final result was also interpreted as OR and 95% CI as in a logistic regression.

All statistical analyses were performed using R 4.2.1. The packages used in this study include “CBPS”, “MatchIt”, “WeightIt”, “geepack”, and “survival”.

### Role of funding source

The study is not supported by any funding.

## Results

After excluding patients without valid EHR, a total of 112,334 patients were included in the final analysis. Among them, 111,442 patients had no SCT/SCD, while 504 patients had SCT and 388 SCD. Table 1 shows the distributions of demographic characteristics, comorbidities, and COVID-19-related outcomes among the original study population. Compared to the general US population from NHANES, the patient population at UCM had similar distributions in age and smoking history. However, non-Hispanic Black people were more representative in the patient population at UCM; moreover, we observed more female in the patient population. The non-Hispanic Black people is the largest racial group, accounting for more than 45% of the total population and a much higher proportion (>94%) of patients with SCT/SCD. When compared to patients without SCT/SCD, patients with SCT/SCD were also more likely to be younger (age ≤35: 33.8% vs. 57.1/55.2%), female (58.7% vs. 88.5/62.9%), and non-smokers (42.1% vs. 65.1/56.2%). Among the eight comorbidities included in this study, patients with SCT/SCD had higher prevalence of chronic kidney disease (8.9% vs. 11.9/14.4%), asthma (8.1% vs. 24.8/27.8%), and heart failure (7.3% vs. 8.1/15.7%), while no substantial difference was observed for other comorbidities.

**Table 1:** Distributions of demographic characteristics, comorbidities, and COVID-19-related outcomes among study population according to SCT/SCD status.

	Patients without SCT/SCD	Patients with only SCT	Patients with only SCD	NHANES 2017 to march 2020 pre-pandemic^a^
	(*n* = 1,11,442)	(*n* = 504)	(*n* = 388)	
Demographic characteristics				
Age				
≤35	37,640 (33.8)	288 (57.1)	214 (55.2)	30.1
36–50	23,283 (20.9)	122 (24.2)	108 (27.8)	24.4
51–65	26,380 (23.7)	51 (10.1)	44 (11.3)	26.0
>65	24,139 (21.7)	43 (8.5)	22 (5.7)	19.5
BMI group				
<18.5	2,141 (1.9)	12 (2.4)	35 (9.0)	11.3
18.5–25	24,853 (22.3)	113 (22.4)	176 (45.4)	26.9
25.1–30	24,325 (21.8)	114 (22.6)	82 (21.1)	25.7
30.1–40	24,758 (22.2)	180 (35.7)	73 (18.8)	25.6
>40	7,847 (7.0)	68 (13.5)	6 (1.6)	6.9
Missing	27,518 (24.7)	17 (3.4)	16 (4.1)	3.7
Race				
Non-hispanic white	32,073 (28.8)	4 (0.8)	2 (0.5)	59.4
Non-hispanic black	52,765 (47.4)	475 (94.3)	378 (97.4)	12.0
Hispanic	6,473 (5.8)	10 (2.0)	3 (0.8)	18.3
Other	5,768 (5.2)	15 (3.0)	5 (1.3)	10.3
Missing/unknown	14,363 (12.9)	0 (0)	0 (0)	0
Sex				
Female	65,415 (58.7)	446 (88.5)	244 (62.9)	48.9
Male	45,994 (41.3)	58 (11.5)	144 (37.1)	51.1
Missing/unknown	33 (0.0)	0 (0)	0 (0)	0
Smoking history				
Never	46,886 (42.1)	328 (65.1)	218 (56.2)	45.0
Current or passive smoking	10,356 (9.3)	56 (11.1)	64 (16.5)	12.6
Quit smoking	20,630 (18.5)	88 (17.5)	60 (15.5)	19.3
Missing	33,570 (30.1)	32 (6.4)	46 (11.9)	23.1
Comorbidities				
Hypertension				
No	80,514 (72.3)	351 (69.6)	273 (70.4)	
Yes	30,928 (27.8)	153 (30.4)	115 (29.6)	
Type 2 diabetes				
No	96,001 (86.1)	418 (82.9)	350 (90.2)	
Yes	15,441 (13.9)	86 (17.1)	38 (9.8)	
Chronic kidney disease				
No	1,01,548 (91.1)	444 (88.1)	332 (85.6)	
Yes	9,894 (8.9)	60 (11.9)	56 (14.4)	
Heart failure				
No	1,03,268 (92.7)	463 (91.9)	327 (84.3)	
Yes	8,174 (7.3)	41 (8.1)	61 (15.7)	
Asthma				
No	1,02,461 (91.9)	379 (75.2)	280 (72.2)	
Yes	8,981 (8.1)	125 (24.8)	108 (27.8)	
Chronic obstructive pulmonary disease				
No	1,05,850 (95.0)	483 (95.8)	369 (95.1)	
Yes	5,592 (5.0)	21 (4.2)	19 (4.9)	
Chronic ischemic heart disease				
No	1,01,947 (91.5)	470 (93.3)	350 (90.2)	
Yes	9,495 (8.5)	34 (6.8)	38 (9.8)	
Cerebral infarction				
No	1,09,017 (97.8)	498 (98.8)	371 (95.6)	
Yes	2,425 (2.2)	6 (1.2)	17 (4.4)	
COVID-19 outcomes				
Ever COVID-19 positive				
No	96,203 (86.3)	421 (83.5)	320 (82.5)	
Yes	15,239 (13.7)	83 (16.5)	68 (17.5)	
COVID-19-related ICU admission				
No	1,10,228 (98.9)	500 (99.2)	375 (96.7)	
Yes	1,214 (1.1)	4 (0.8)	13 (3.4)	
COVID-19-related mortality				
No	1,10,931 (99.5)	503 (99.8)	385 (99.2)	
Yes	511 (0.5)	1 (0.2)	3 (0.8)	
COVID-19-related ARDS				
No	1,10,008 (98.7)	492 (97.6)	374 (96.4)	
Yes	1,434 (1.3)	12 (2.4)	14 (3.6)	
COVID-19-related pneumonia/ACS				
No	1,09,210 (98.0)	485 (96.2)	364 (93.8)	
Yes	2,232 (2.0)	19 (3.8)	24 (6.2)	
COVID-19-related pain				
No	1,09,508 (98.3)	486 (96.4)	348 (89.7)	
Yes	1,934 (1.7)	18 (3.6)	40 (10.3)	
COVID-19-related shortness of breath				
No	1,09,790 (98.5)	491 (97.4)	379 (97.7)	
Yes	1,652 (1.5)	13 (2.6)	9 (2.3)	
COVID-19-related VTE/PE				
No	1,11,103 (99.7)	501 (99.4)	384 (99.0)	
Yes	339 (0.3)	3 (0.6)	4 (1.0)	

ACS, acute chest syndrome; ARDS, acute respiratory distress syndrome; PE, pulmonary embolism; SCD, sickle cell disease; SCT, sickle cell trait; VTE, venous thromboembolism.

^a^ Only weighted percentages are shown for NHANES 2017 to march 2020 pre-pandemic, which represents the general US population.

**Figure 1:**
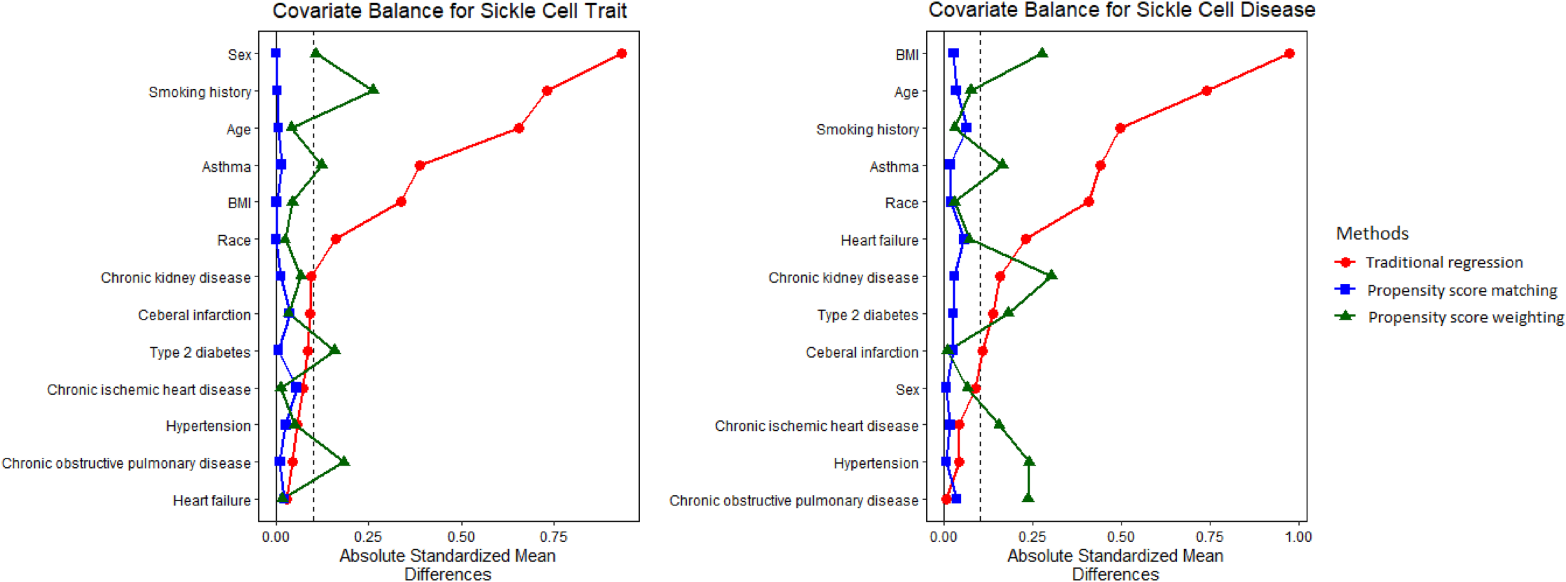
Absolute standardized mean difference (ASMD) for original, matched, and weighted populations. ASMD values <0.1 indicate good covariate balance, strengthening the interpretability and validity of our analyses as providing evidence of causality.

Distributions of covariates using CBPS matching and weighting can be found in Supplementary Tables S1, S2. Substantial difference was not observed for demographic characteristics or preexisting comorbidities between study populations with and without SCT/SCD. For instance, the prevalence of asthma, which demonstrated huge difference in the original population, was comparable for both SCT (CBPS matching: 24.2% vs. 24.8%; CBPS weighting: 24.8% vs. 24.8%) and SCD (CBPS matching: 27.1% vs. 27.8%; CBPS weighting: 27.2% vs. 27.8%). It is clear that the causal inference approaches generated a study population with more balanced distributions of covariates for analysis. The covariate balance that was measured as ASMD in this study indicates the quality of approaches at recovering randomized experiments and inform the degree to which we can make a valid causal assessment. Figure 1 shows that ASMD was smaller than 0.1 in most covariates when using causal inference methods, especially CBPS matching, thus strengthening the interpretability and validity of our analyses as providing evidence of causality.

**Figure 2:**
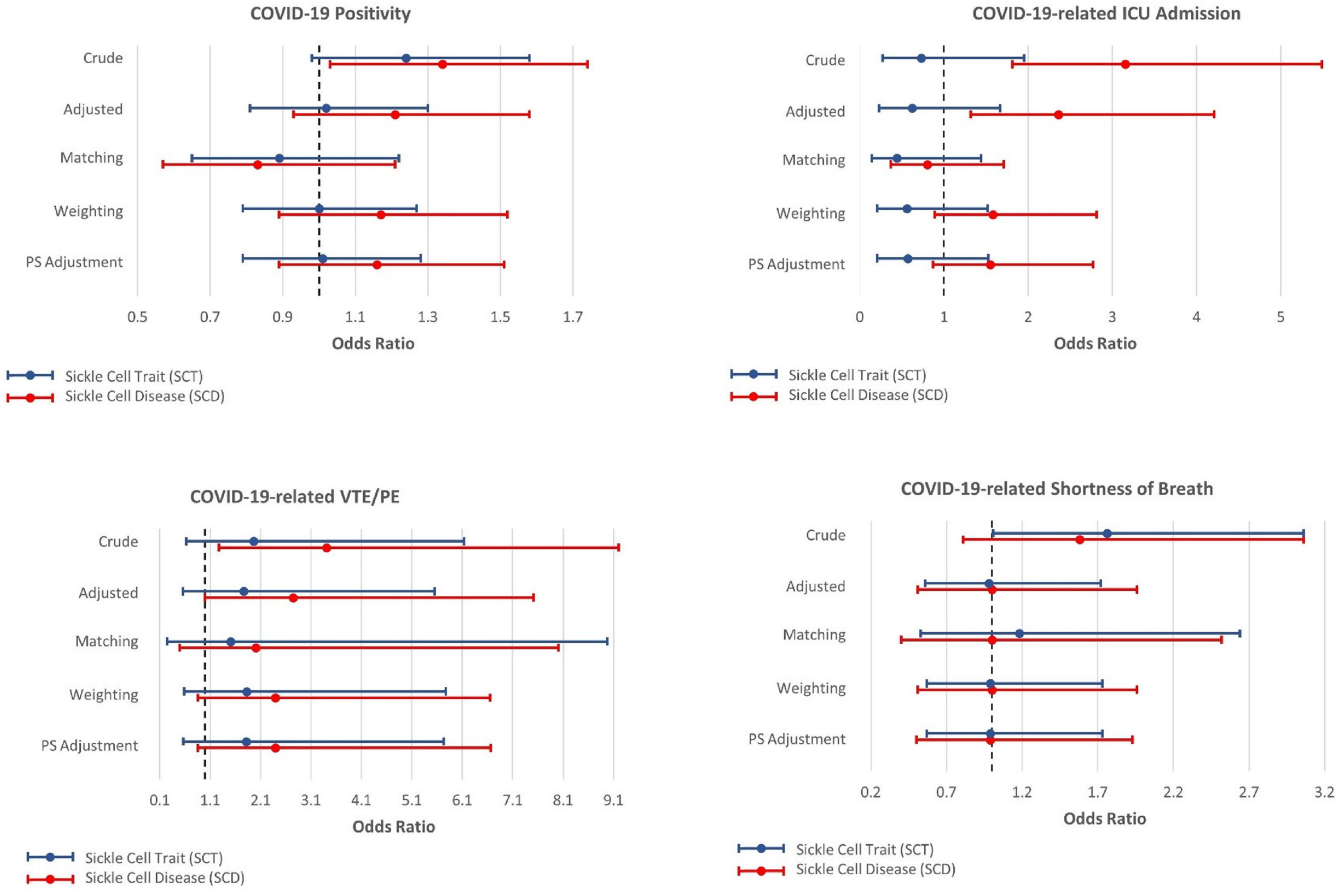
Odds ratios and 95% confidence interval for sickle cell trait and disease in relation to COVID-19-related positivity, ICU admission, venous thromboembolism, and pulmonary embolism (VTE/PE), and shortness of breath, which exhibit no association with SCT/SCD in the causal inference approaches.

**Figure 3:**
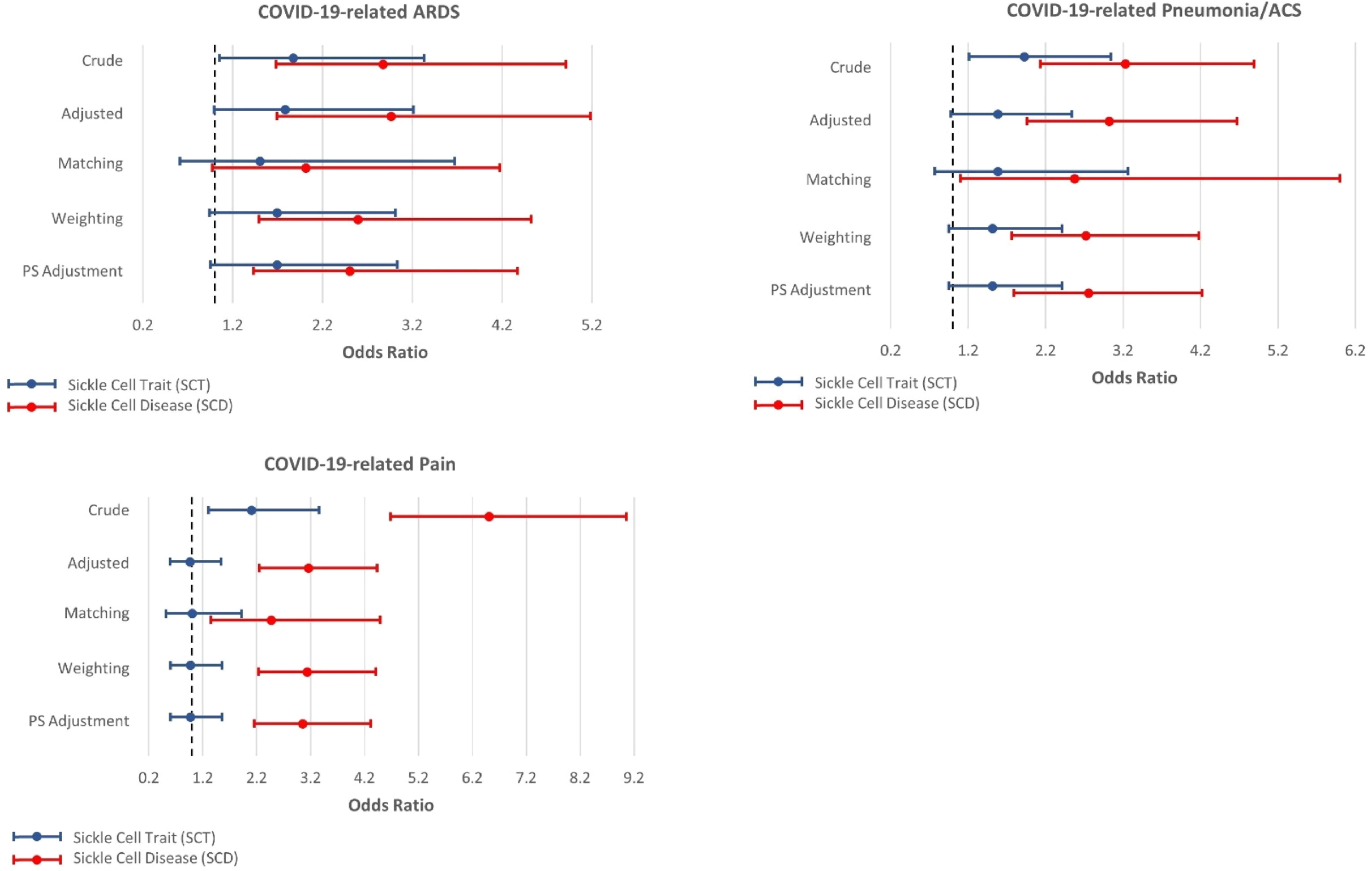
Odds ratios and 95% confidence interval for sickle cell trait and disease in relation to COVID-19-related acute respiratory distress syndrome (ARDS), pneumonia or acute chest syndrome (ACS), and pain, which exhibit associations with SCT/SCD in the causal inference approaches.

Figures 2, 3 present the associations between SCT/SCD and COVID-19-related outcomes. Figure 2 presents outcomes that exhibited no association in causal inference approaches, including COVID-19 positivity, ICU admission, VTE/PE, and shortness of breath, while Figure 3 presents outcomes with significant associations in causal inference approaches, including pneumonia/ACS, pain, and ARDS. The specific values can be found in Supplementary Table S3. COVID-19-related mortality was not analyzed in this study because of limited sample size.

In unadjusted models, SCD were associated with most COVID-19-related outcomes, including COVID-19 positivity (OR = 1.34, 95% CI: 1.03–1.74), ICU admission (OR = 3.15, 95% CI: 1.81–5.49), ARDS (OR = 2.87, 95% CI: 1.68–4.91), pneumonia/ACS (OR = 3.23, 95% CI: 2.13–4.89), pain (OR = 6.51, 95% CI: 4.68–9.06), and VTE/PE (OR = 3.41, 95% CI: 1.27–9.20). In comparison, SCT was not associated with COVID-19 positivity, ICU admission, and VTE/PE, though still associated with ARDS (OR = 1.87, 95% CI: 1.05–3.33), pneumonia/ACS (OR = 1.92, 95% CI: 1.21–3.04), and pain (OR = 2.10, 95% CI: 1.31–3.36) in unadjusted traditional models. As anticipated, these associations observed in unadjusted models became attenuated or even null in other approaches, particularly for SCT.

All other four approaches provided generally consistent results, though some divergence was observed across these approaches. The traditional regression model adjusted for covariates provided the strongest associations, while causal inference approaches, particularly CBPS matching, yielded more conservative estimates. SCT was no longer associated with any COVID-19-related outcomes, though some suggestive associations were observed in three approaches including traditional adjusted regression model, CBPS weighting, and CBPS adjustment, for ARDS (OR estimates, from 1.78 (95% CI: 0.99–3.21) to 1.69 (95% CI: 0.94–3.01)) and pneumonia/ACS (OR estimates, from 1.58 (95% CI: 0.98–2.54) to 1.51 (95% CI: 0.95–2.41)). However, these associations were not supported by CBPS matching approach (ARDS: OR = 1.50, 95% CI: 0.61–3.67; pneumonia/ACS: OR = 1.58, 95% CI: 0.77–3.26).

On the other hand, in all four approaches, SCD was associated with pneumonia/ACS (OR estimates, from 3.03 (95% CI: 1.96–4.67) to 2.57 (95% CI: 1.10–6.00)), and pain (OR estimates, from 3.16 (95% CI: 2.25–4.43) to 2.47 (1.35–4.49)). While CBPS matching only suggested a possible association between SCD and ARDS (OR = 2.01, 95% CI: 0.97–4.17), this association was statistically significant in other three approaches (OR estimates, from 2.96 (95% CI: 1.69–5.18) to 2.50 (95% CI: 1.43–4.37)). Moreover, the traditional regression model adjusted for covariates presented associations of SCD with ICU admission (OR = 2.36, 95% CI: 1.32–4.21) and VTE/PE (OR = 2.74, 95% CI: 1.00–7.51), however, these associations were not corroborated in causal inference approaches. COVID-19 positivity, which exhibited an association with SCD in unadjusted analysis, was no longer associated with SCD in these approaches.

## Discussion

This study provides robust and reproducible evidence on the link between SCT/SCD status and COVID-19-related outcomes. In light of the very large patient population, the multiple approaches employed in this study, and the balanced distributions of covariates in demographic characteristics and comorbidities, we conclude that SCD status is linked to worse COVID-19 outcomes, with the strongest evidence for pneumonia/ACS, pain, and ARDS. These results were robust across numerous approaches and models in this study.

Our results suggest that patients with SCT have similar COVID-19-related outcomes compared to patients without SCT/SCD. These findings are consistent with the consensus that SCT is mainly asymptomatic and confirm conclusions from recent studies that SCT is not associated with worse COVID-19 outcomes^31^. It is noteworthy that the majority of patients with SCT were female, even a higher proportion than that in patients with SCD, possibly due to SCT test at the time of obstetrics/gynecology care. The high proportion of female patients in this cohort aligns with observations in another recent SCT/SCD and COVID-19 study (25), suggesting that the knowledge of SCT status is limited to a subset of patients. Prior studies have raised the same concern as researchers found that ICD codes for SCT were highly specific but with a low sensitivity (26). The overrepresentation of female among patients with SCT also poses challenge to studies that investigate the clinical outcomes associated with SCT, as the unbalanced distribution of demographic characteristics could induce confounding. Moreover, these patients who are aware of their SCT carrier status may be more likely to seek medical assistance, thus leading to modified effect of SCT among this particular population when compared to patients unaware of their SCT status. A few prior studies fail to consider these confounding and effect modification, thus concluding that patients with SCT also have increased risks for COVID-19-related outcomes, such as hospitalization and mortality (27, 28). In this case, causal inference approaches demonstrate advantages over traditional regression methods, because causal inference approaches aim to recover randomized experiments through constructing pseudo-populations with balanced distributions of all available covariates, thus eliminating potential confounding and effect modification, and providing evidence for causality, while traditional regression methods fail to incorporate any consideration of the unbalanced covariate distribution. Therefore, results for SCT from causal inference approaches provide additional, stronger evidence for the association between SCT and COVID-19 outcomes.

SCD, on the other hand, is associated with worse COVID-19 outcomes in our findings. Consistent evidence was reported for pneumonia/ACS and COVID-19-related pain across all four approaches in this study, while three out of four approaches also supported an association with ARDS. ACS and pain are established comorbidities associated with SCD as well as COVID-19 (29). Moreover, a prior study suggested that both ACS and pain are independent risk factors for worse COVID-19 outcomes (30). Our findings suggest a synergistic interaction between SCD and COVID-19, as we observed the strongest association between SCD and COVID-19-related pain. Notably, this association was not observed in SCT patients, suggesting that SCD and COVID-19 synergistically lead to worse ACS and pain.

ARDS was less frequently investigated in cohort studies on the association between SCD and COVID-19-related outcomes, though some case studies reported development of ARDS among patients with SCD after COVID-19 infection (28). In this study, even the most conservative approach, CBPS matching, illustrates an association between SCD and COVID-19-related ARDS, which is consistent with prior case reports. VTE/PE is another major concern, which is prevalent in both patients with SCD and COVID-19 (31, 32). Therefore, having both SCD and COVID-19 might result in severe VTE/PE as hypothesized in prior studies (28). However, in this study, although we observed a significant association between SCD and COVID-19-related VTE/PE using traditional regression methods, this association was not corroborated in causal inference approaches. The divergence may be explained by the advantage of causal inference approaches to deal with the unbalanced distribution of preexisting conditions. Prior studies have pointed out that preexisting conditions, such as obesity type 2 diabetes, and hypertension, contributes to the development of VTE/PE (33). Patients with SCD are more likely to have these conditions, as evidenced by our study population. Traditional regression was unable to address the overrepresentation of these conditions among SCD patients. Therefore, the significant association in traditional regression was a result of confounding of these conditions, rather than SCD. However, the null association of VTE/PE could also be a consequence of limited VTE/PE cases. In this cohort, only a few patients with SCT/SCD had diagnosis of COVID-19-related PTE/VE. The limited sample size could reduce statistical power and thus prevent us from reaching the true conclusion between SCD and COVID-19-related VTE/PE. Overall, the associations of VTE/PE in our findings warrant future investigation. In general, our findings from this study highlight the importance of COVID-19 prevention among patients with SCD, a particularly vulnerable population.

COVID-19-related mortality is a widely investigated outcome in prior studies (28). As mentioned by a recent review, although several studies concluded that there is an elevated association between SCD and mortality after COVID-19 infection, cohort studies that utilize a matching method for preexisting conditions reported no difference in COVID-19-related mortality between patients with and without SCD (28). This example illustrates the importance of considering the unbalanced distribution of demographic characteristics and preexisting medical conditions when investigating the impact of SCD. Unfortunately, our study did not have a sufficiently large sample size to analyze COVID-19-related mortality. However, other outcomes included in this study provide complementary evidence to the adverse effect of SCD after COVID-19 infection, and illustrate the effectiveness of causal inference approaches that disentangle the medical condition of main interest from other associated comorbidities.

The current study has some limitations. First, this is not a multi-institution study so generalizability may be limited. All the patients are from UCM and thus our analysis may also be subject to potential systematic bias. Second, the study population was restricted to patients who visited UCM for medical care, presenting potential selection bias. However, the potential selection bias only leads to an underestimation of true associations between SCT/SCD and COVID-19-related outcomes. Given that we have already observed some associations in this study, this limitation would only strengthen our concerns over COVID-19 infection among patients with SCD. Third, our analyses did not account for secular trends of the pandemic and treatments, which might influence outcomes. New COVID-19 variants have evolved during the pandemic and pose distinct harms to patients. Treatment strategies and vaccines have also been developed over the study time frame to alleviate disease burdens cause by COVID-19. A reasonable hypothesis could be that patients with SCD experienced less severe COVID-19-related outcomes later in the study compared to patients at the early stage of the pandemic. These secular changes should be considered in future studies. Finally, no pediatric patients were included in this study. SCT/SCD among pediatric patients is a crucial research topic and these patients may also manifest distinct outcomes after COVID-19 infection. Therefore, our conclusions may not be generalizable to pediatric patients.

In summary, data in this study provide evidence that SCD imposes additional risk of severe COVID-19-related outcomes, particularly pneumonia/ACS, pain, and ARDS, after balancing for demographic characteristics and other preexisting conditions using multiple causal inference approaches. Though some associations were observed for SCT in traditional regression methods, we found no evidence to support these associations in causal inference approaches. The study has highlighted the effectiveness of causal inference approaches when investigating the impacts of SCD/SCT and provide more evidence for the link between SCD/SCT and COVID-19-related outcomes.

## Data Availability

The data analyzed in this study is subject to the following licenses/restrictions: The data will be available upon reasonable request. Requests to access these datasets should be directed to jjohnso3@bsd.uchicago.edu.
