# Supplementary material for: Evaluating the impact of sickle cell disease on COVID-19 susceptibility and severity: a retrospective cohort study based on electronic health record
Source: Front Epidemiol. 2023 Sep 12;3:1241645. doi: 10.3389/fepid.2023.1241645 (PMC10910923; doi:10.3389/fepid.2023.1241645)
Supplement: Supplementary file 1 [file Datasheet1.docx]

**Title: Evaluating the impact of sickle cell disease on COVID-19 susceptibility and severity: an application of causal inference methods – supplemental materials**

**S.1 Supplemental methods**

**S.1.1 Absolute standardized mean difference**

Absolute standardized mean difference (ASMD) is one of the most common metrics to measure covariate balance. Similar to the effect size, the ASMD is calculated as the absolute value in the difference in means of a covariate across the treatment groups, divided by the standard deviation in the treated group. The ASMD has been extended to categorical variables as discussed by Stuart et al^1^. Guidelines indicate that 0.1 or 0.25 represent reasonable cutoffs for acceptable ASMD's larger standardized biases indicate that groups are too different from one another for reliable comparison.

**S.1.2 Propensity score matching approach**

In this study, we conduct a 1 to 1 matching based on the covariate balancing propensity score (CBPS). Matching exactly on the CBPS is typically impossible. The nearest-neighbor matching algorithm is the most commonly used method. However, the nearest-neighbor matching is essentially “greedy” that there is no action taken to optimize the global matching performance and each match is selected without considering the other matches that may occur subsequently.

To avoid these limitations, we chose to use optimal matching algorithm that minimize the absolute pairwise distance in the matched sample. Advantages of the optimal matching algorithm include that the matching order is not required to be specified and it is less likely that there would be extremely large within-pair distances, which may be observed in nearest-neighbor matching.^2^

Logistic regression can take matching into consideration by including a constant term for each pair. However, since the participants are 1:1 matched, the number of parameters grow dramatically, which leads to hundreds of more parameter in the model. In this case, the conventional logistic regression based on maximum likelihood estimation is not valid and the estimation is biased.

To address this challenge, the conditional logistic regression used for matching is proposed^3^. This approach deals with this challenge by conditioning out, instead of estimating, the pair-specific constants. In other words, the conditional logistic regression is based on maximizing the conditional likelihood. Though we cannot obtain the intercept estimates for each pair from conditional logistic regression, we can still obtain the slope estimate, which is the parameter of interest.

**S.1.3 Propensity score weighting approach**

A weight is generated for each participant using the inverse of CBPS calculated in the prior step. Most studies usually stabilize the weights by multiplying the weight with the marginal probabilities of the exposure, aiming to reduce estimation variance and avoid extremely large weights. The stabilized and unstabilized weights also yield distinct confidence intervals in the traditional regression models.

To compute a consistent confidence interval, we decide to use the robust variance estimator. We fit generalized estimating equation (GEE) models with an independent working correlation among participants in the weighted pseudo-population. This approach gives identical results for both stabilized and unstabilized weights. Therefore, we did not differentiate between stabilized and unstabilized weights in this study. It should be noted that the confidence interval based on the robust variance estimator is valid, but also conservative^4^.

**S.1.4 Propensity score adjustment approach**

Hirano and Imbens show that inclusion of propensity score as a covariate in the model can remove confounding bias when estimating the causal effect. However, the causal interpretation is based on the counterfactuals predicted by the model. The procedure is described below.

First, model the outcome given the exposure and estimated propensity score. Second, output the coefficient estimates from the regression model, and use these estimates to calculate the predicted outcomes (i.e., counterfactual outcomes) when the value of the exposure of interest (in our case, SCT or SCD) is set to 0 and 1 for all participants, respectively. The causal effect then can be estimated using these counterfactual outcomes. The 95% confidence interval can be computed using bootstrapping.

**S.2 Supplemental tables**

Table S1. Distributions of demographic characteristics, comorbidities, and COVID-19-related outcomes among matched populations after propensity score matching

|  | Matched patients for SCT  (n=504) | Patients with only SCT  (n=504) | Matched patients for SCD  (n=388) | Patients with only SCD  (n=388) |
| --- | --- | --- | --- | --- |
| **Demographic characteristics** | |  |  |  |
| Age |  |  |  |  |
| ≤35 | 288 (57.1) | 288 (57.1) | 208 (53.6) | 214 (55.2) |
| 36-50 | 122 (24.2) | 122 (24.2) | 110 (28.4) | 108 (27.8) |
| 51-65 | 54 (10.7) | 51 (10.1) | 46 (11.9) | 44 (11.3) |
| >65 | 40 (7.9) | 43 (8.5) | 24 (6.2) | 22 (5.7) |
| BMI group |  |  |  |  |
| <18.5 | 12 (2.4) | 12 (2.4) | 38 (9.8) | 35 (9.0) |
| 18.5-25 | 115 (22.8) | 113 (22.4) | 173 (44.6) | 176 (45.4) |
| 25.1-30 | 110 (21.8) | 114 (22.6) | 85 (21.9) | 82 (21.1) |
| 30.1-40 | 183 (36.3) | 180 (35.7) | 73 (18.8) | 73 (18.8) |
| >40 | 67 (13.3) | 68 (13.5) | 6 (1.5) | 6 (1.6) |
| Missing | 17 (3.4) | 17 (3.4) | 13 (3.4) | 16 (4.1) |
| Race |  |  |  |  |
| Non-Hispanic White | 4 (0.8) | 4 (0.8) | 2 (0.5) | 2 (0.5) |
| Non-Hispanic Black | 475 (94.2) | 475 (94.3) | 379 (97.7) | 378 (97.4) |
| Hispanic | 10 (2.0) | 10 (2.0) | 3 (0.8) | 3 (0.8) |
| Other | 15 (3.0) | 15 (3.0) | 4 (1.0) | 5 (1.3) |
| Missing/unknown | 4 (0.8) | 0 (0) | 2 (0.5) | 0 (0) |
| Sex |  |  |  |  |
| Female | 446 (88.5) | 446 (88.5) | 243 (62.6) | 244 (62.9) |
| Male | 58 (11.5) | 58 (11.5) | 145 (37.4) | 144 (37.1) |
| Missing/unknown | 0 (0) | 0 (0) | 0 (0) | 0 (0) |
| Smoking history |  |  |  |  |
| Never | 328 (65.1) | 328 (65.1) | 205 (52.8) | 218 (56.2) |
| Current or passive smoking | 56 (11.1) | 56 (11.1) | 66 (17.0) | 64 (16.5) |
| Quit smoking | 89 (17.7) | 88 (17.5) | 68 (17.5) | 60 (15.5) |
| Missing | 31 (6.2) | 32 (6.4) | 49 (12.6) | 46 (11.9) |
|  |  |  |  |  |
| **Comorbidities** |  |  |  |  |
| Hypertension |  |  |  |  |
| No | 357 (70.8) | 351 (69.6) | 274 (70.6) | 273 (70.4) |
| Yes | 147 (29.2) | 153 (30.4) | 114 (29.4) | 115 (29.6) |
| Type 2 diabetes |  |  |  |  |
| No | 419 (83.1) | 418 (82.9) | 347 (89.4) | 350 (90.2) |
| Yes | 85 (16.9) | 86 (17.1) | 41 (10.6) | 38 (9.8) |
| Chronic kidney disease |  |  |  |  |
| No | 446 (88.5) | 444 (88.1) | 328 (84.5) | 332 (85.6) |
| Yes | 58 (11.5) | 60 (11.9) | 60 (15.5) | 56 (14.4) |
| Heart failure |  |  |  |  |
| No | 466 (92.5) | 463 (91.9) | 319 (82.2) | 327 (84.3) |
| Yes | 38 (7.5) | 41 (8.1) | 69 (17.8) | 61 (15.7) |
| Asthma |  |  |  |  |
| No | 382 (75.8) | 379 (75.2) | 283 (72.9) | 280 (72.2) |
| Yes | 122 (24.2) | 125 (24.8) | 105 (27.1) | 108 (27.8) |
| Chronic obstructive pulmonary disease | |  |  |  |
| No | 484 (96.0) | 483 (95.8) | 372 (95.9) | 369 (95.1) |
| Yes | 20 (4.0) | 21 (4.2) | 16 (4.1) | 19 (4.9) |
| Chronic ischemic heart disease | |  |  |  |
| No | 463 (91.9) | 470 (93.3) | 352 (90.7) | 350 (90.2) |
| Yes | 41 (8.1) | 34 (6.8) | 36 (9.3) | 38 (9.8) |
| Cerebral infarction |  |  |  |  |
| No | 500 (99.2) | 498 (98.8) | 373 (96.1) | 371 (95.6) |
| Yes | 4 (0.8) | 6 (1.2) | 15 (3.9) | 17 (4.4) |
|  |  |  |  |  |
| **COVID-19 outcomes** |  |  |  |  |
| Ever COVID-19 positive |  |  |  |  |
| No | 412 (81.7) | 421 (83.5) | 310 (79.9) | 320 (82.5) |
| Yes | 92 (18.3) | 83 (16.5) | 78 (90.1) | 68 (17.5) |
| COVID-19-related ICU admission | |  |  |  |
| No | 495 (98.2) | 500 (99.2) | 372 (95.9) | 375 (96.7) |
| Yes | 9 (1.8) | 4 (0.8) | 16 (4.1) | 13 (3.4) |
| COVID-19-related mortality |  |  |  |  |
| No | 503 (99.8) | 503 (99.8) | 386 (99.5) | 385 (99.2) |
| Yes | 1 (0.2) | 1 (0.2) | 2 (0.5) | 3 (0.8) |
| COVID-19-related ARDS |  |  |  |  |
| No | 496 (98.4) | 492 (97.6) | 377 (97.2) | 374 (96.4) |
| Yes | 8 (1.6) | 12 (2.4) | 11 (2.8) | 14 (3.6) |
| COVID-19-related pneumonia/ACS | |  |  |  |
| No | 492 (97.6) | 485 (96.2) | 372 (95.9) | 364 (93.8) |
| Yes | 12 (2.4) | 19 (3.8) | 16 (4.1) | 24 (6.2) |
| COVID-19-related pain |  |  |  |  |
| No | 486 (96.4) | 486 (96.4) | 370 (95.4) | 348 (89.7) |
| Yes | 18 (3.6) | 18 (3.6) | 18 (4.6) | 40 (10.3) |
| COVID-19-related shortness of breath | |  |  |  |
| No | 493 (97.8) | 491 (97.4) | 379 (97.7) | 379 (97.7) |
| Yes | 11 (2.2) | 13 (2.6) | 9 (2.3) | 9 (2.3) |
| COVID-19-related VTE/PE | |  |  |  |
| No | 502 (99.6) | 501 (99.4) | 384 (99.0) | 384 (99.0) |
| Yes | 2 (0.4) | 3 (0.6) | 4 (1.0) | 4 (1.0) |
| ACS, acute chest syndrome; ARDS, acute respiratory distress syndrome; PE, pulmonary embolism; SCD, sickle cell disease; SCT, sickle cell trait; VTE, venous thromboembolism. | | | | |

Table S2. Distributions (percentages) of demographic characteristics, comorbidities, and COVID-19-related outcomes among pseudo-populations after propensity score weighting.

|  | Weighted comparison patients for SCT | Weighted patients with only SCT | Weighted comparison patients for SCD | Weighted patients with only SCD |
| --- | --- | --- | --- | --- |
| **Demographic characteristics** | |  |  |  |
| Age |  |  |  |  |
| ≤35 | 57.1 | 57.1 | 55.2 | 55.2 |
| 36-50 | 24.2 | 24.2 | 27.8 | 27.8 |
| 51-65 | 10.1 | 10.1 | 11.3 | 11.3 |
| >65 | 8.5 | 8.5 | 5.7 | 5.7 |
| BMI group |  |  |  |  |
| <18.5 | 2.4 | 2.4 | 8.4 | 9.0 |
| 18.5-25 | 22.4 | 22.4 | 45.9 | 45.4 |
| 25.1-30 | 22.6 | 22.6 | 21.6 | 21.1 |
| 30.1-40 | 35.7 | 35.7 | 17.8 | 18.8 |
| >40 | 13.5 | 13.5 | 1.8 | 1.6 |
| Missing | 3.4 | 3.4 | 4.5 | 4.1 |
| Race |  |  |  |  |
| Non-Hispanic White | 0.8 | 0.8 | 0.5 | 0.5 |
| Non-Hispanic Black | 94.2 | 94.2 | 97.4 | 97.4 |
| Hispanic | 2.0 | 2.0 | 0.8 | 0.8 |
| Other | 3.0 | 3.0 | 1.3 | 1.3 |
| Missing/unknown | 0 | 0 | 0 | 0 |
| Sex |  |  |  |  |
| Female | 88.5 | 88.5 | 62.9 | 62.9 |
| Male | 11.5 | 11.5 | 37.1 | 37.1 |
| Missing/unknown | 0 | 0 | 0 | 0 |
| Smoking history |  |  |  |  |
| Never | 64.7 | 65.1 | 56.2 | 56.2 |
| Current or passive smoking | 10.2 | 11.1 | 16.5 | 16.5 |
| Quit smoking | 16.9 | 17.5 | 15.5 | 15.5 |
| Missing | 6.2 | 6.3 | 11.8 | 11.8 |
|  |  |  |  |  |
| **Comorbidities** |  |  |  |  |
| Hypertension |  |  |  |  |
| No | 69.6 | 69.6 | 71.0 | 70.4 |
| Yes | 30.4 | 30.4 | 29.0 | 29.6 |
| Type 2 diabetes |  |  |  |  |
| No | 82.2 | 82.9 | 89.8 | 90.2 |
| Yes | 17.8 | 17.1 | 10.2 | 9.8 |
| Chronic kidney disease |  |  |  |  |
| No | 88.1 | 88.1 | 86.6 | 85.6 |
| Yes | 11.9 | 11.9 | 13.4 | 14.4 |
| Heart failure |  |  |  |  |
| No | 91.9 | 91.9 | 84.3 | 84.3 |
| Yes | 8.1 | 8.1 | 15.7 | 15.7 |
| Asthma |  |  |  |  |
| No | 75.2 | 75.2 | 72.8 | 72.2 |
| Yes | 24.8 | 24.8 | 27.2 | 27.8 |
| Chronic obstructive pulmonary disease | |  |  |  |
| No | 95.4 | 95.8 | 94.2 | 95.1 |
| Yes | 4.6 | 4.2 | 5.8 | 4.9 |
| Chronic ischemic heart disease | |  |  |  |
| No | 93.5 | 93.2 | 91.3 | 90.2 |
| Yes | 6.5 | 6.8 | 8.7 | 9.8 |
| Cerebral infarction |  |  |  |  |
| No | 98.8 | 98.8 | 95.6 | 95.6 |
| Yes | 1.2 | 1.2 | 4.4 | 4.4 |
|  |  |  |  |  |
| **COVID-19 outcomes** |  |  |  |  |
| Ever COVID-19 positive |  |  |  |  |
| No | 83.6 | 83.5 | 84.6 | 82.5 |
| Yes | 16.4 | 16.5 | 15.4 | 17.5 |
| COVID-19-related ICU admission | |  |  |  |
| No | 98.6 | 99.2 | 97.9 | 96.6 |
| Yes | 1.4 | 0.8 | 2.1 | 3.4 |
| COVID-19-related mortality |  |  |  |  |
| No | 98.0 | 98.0 | 96.8 | 97.2 |
| Yes | 2.0 | 2.0 | 3.2 | 2.8 |
| COVID-19-related ARDS |  |  |  |  |
| No | 98.6 | 97.6 | 98.6 | 96.4 |
| Yes | 1.4 | 2.4 | 1.4 | 3.6 |
| COVID-19-related pneumonia/ACS | |  |  |  |
| No | 97.5 | 96.2 | 97.6 | 93.8 |
| Yes | 2.5 | 3.8 | 2.4 | 6.2 |
| COVID-19-related pain |  |  |  |  |
| No | 96.3 | 96.4 | 96.5 | 89.7 |
| Yes | 3.7 | 3.6 | 3.5 | 10.3 |
| COVID-19-related shortness of breath | |  |  |  |
| No | 97.4 | 97.4 | 97.7 | 97.7 |
| Yes | 2.6 | 2.6 | 2.3 | 2.3 |
| COVID-19-related VTE/PE | |  |  |  |
| No | 99.7 | 99.4 | 99.6 | 99.0 |
| Yes | 0.3 | 0.6 | 0.4 | 1.0 |
| Only percentages are shown for the weighted pseudo-population, as the frequency has no meaning for the pseudo-population.  ACS, acute chest syndrome; ARDS, acute respiratory distress syndrome; PE, pulmonary embolism; SCD, sickle cell disease; SCT, sickle cell trait; VTE, venous thromboembolism. | | | | |

Table S3. Odds ratios and 95% confidence interval for SCT and SCD status in relation to COVID-19 related outcomes

|  | **COVID-19 positivity** | **ICU admission** | **ARDS** | **Pneumonia/ACS** | **Pain** | **Shortness of breath** | **VTE/PE** |
| --- | --- | --- | --- | --- | --- | --- | --- |
| **SCT** |  |  |  |  |  |  |  |
| Unadjusted | 1.24 (0.98-1.58) | 0.73 (0.27-1.95) | 1.87 (1.05-3.33) | 1.92 (1.21-3.04) | 2.10 (1.31-3.36) | 1.76 (1.01-3.06) | 1.96 (0.63-6.14) |
| Traditional adjusted model | 1.02 (0.81-1.30) | 0.62 (0.23-1.67) | 1.78 (0.99-3.21) | 1.58 (0.98-2.54) | 0.96 (0.59-1.54) | 0.98 (0.56-1.72) | 1.76 (0.56-5.55) |
| Propensity score matching | 0.89 (0.65-1.22) | 0.44 (0.14-1.44) | 1.50 (0.61-3.67) | 1.58 (0.77-3.26) | 1.00 (0.52-1.92) | 1.18 (0.53-2.64) | 1.50 (0.25-8.98) |
| Propensity score weighting | 1.00 (0.79-1.27) | 0.56 (0.21-1.52) | 1.69 (0.94-3.01) | 1.51 (0.95-2.41) | 0.97 (0.60-1.56) | 0.99 (0.57-1.73) | 1.82 (0.58-5.77) |
| Propensity score adjustment | 1.01 (0.79-1.28) | 0.57 (0.21-1.53) | 1.69 (0.95-3.03) | 1.51 (0.95-2.41) | 0.97 (0.60-1.56) | 0.99 (0.57-1.73) | 1.81 (0.57-5.73) |
|  |  |  |  |  |  |  |  |
| **SCD** |  |  |  |  |  |  |  |
| Unadjusted | 1.34 (1.03-1.74) | 3.15 (1.81-5.49) | 2.87 (1.68-4.91) | 3.23 (2.13-4.89) | 6.51 (4.68-9.06) | 1.58 (0.81-3.06) | 3.41 (1.27-9.20) |
| Traditional adjusted model | 1.21 (0.93-1.58) | 2.36 (1.32-4.21) | 2.96 (1.69-5.18) | 3.03 (1.96-4.67) | 3.16 (2.25-4.43) | 1.00 (0.51-1.96) | 2.74 (1.00-7.51) |
| Propensity score matching | 0.83 (0.57-1.21) | 0.80 (0.37-1.71) | 2.01 (0.97-4.17) | 2.57 (1.10-6.00) | 2.47 (1.35-4.49) | 1.00 (0.40-2.52) | 2.00 (0.50-8.00) |
| Propensity score weighting | 1.17 (0.89-1.52) | 1.58 (0.89-2.81) | 2.59 (1.49-4.52) | 2.72 (1.76-4.18) | 3.13 (2.23-4.41) | 1.00 (0.51-1.96) | 2.39 (0.86-6.65) |
| Propensity score adjustment | 1.16 (0.89-1.51) | 1.55 (0.87-2.77) | 2.50 (1.43-4.37) | 2.75 (1.79-4.22) | 3.05 (2.15-4.32) | 0.99 (0.50-1.93) | 2.39 (0.86-6.66) |
| ACS, acute chest syndrome; ARDS, acute respiratory distress syndrome; PE, pulmonary embolism; SCD, sickle cell disease; SCT, sickle cell trait; VTE, venous thromboembolism. | | | | | | | |

**Reference**

1. Stuart EA, Lee BK, Leacy FP. Prognostic score–based balance measures can be a useful diagnostic for propensity score methods in comparative effectiveness research. *Journal of clinical epidemiology*. 2013;66(8):S84-S90. e1.

2. Hansen BB, Klopfer SO. Optimal full matching and related designs via network flows. *Journal of computational and Graphical Statistics*. 2006;15(3):609-627.

3. Breslow N, Day N, Halvorsen K, Prentice R, Sabai C. Estimation of multiple relative risk functions in matched case-control studies. *American Journal of Epidemiology*. 1978;108(4):299-307.

4. Liang K-Y, Zeger SL. Longitudinal data analysis using generalized linear models. *Biometrika*. 1986;73(1):13-22.
